# Differentiation and management of hepatobiliary mucinous cystic neoplasms: a single centre experience for 8 years

**DOI:** 10.1186/s12893-021-01110-9

**Published:** 2021-03-20

**Authors:** Jiaqi Gao, Junhao Zheng, Jingwei Cai, Mubarak Ali Kirih, Junjie Xu, Liye Tao, Yuelong Liang, Xu Feng, Jing Fang, Xiao Liang

**Affiliations:** Department of General Surgery, School of Medicine, Sir Run Run Shaw Hospital, Zhejiang University, 3 East Qingchun Road, Hangzhou, 310016 Zhejiang China

**Keywords:** Cystadenoma, Liver, Diagnosis, Surgical procedures

## Abstract

**Background:**

Hepatobiliary mucinous cystic neoplasms (H-MCNs) are relatively rare cystic neoplasms in the liver. The differential diagnosis of H-MCNs remains big challenging, and the management and prognosis between the hepatic simple cyst (HSC) and H-MCNs are quite different. This study aimed to present our experience in the management of H-MCNs and provide a preoperative H-MCNs risk prediction nomogram to differentiating H-MCNs from liver cystic lesions.

**Methods:**

29 patients diagnosed with H-MCNs and 75 patients diagnosed with HSC between June 2011 and June 2019 at Zhejiang University School of medicine, Sir Run-Run Shaw Hospital were reviewed in this study. We analyzed the demographic and clinicopathological variables.

**Results:**

US, CT, and MRI could accurately diagnose only 3.4%, 46.1%, and 57.1% of H-MCNs, respectively. After univariate analysis and multivariate logistic regression analysis, the variables significantly associated with H-MCNs were enhancement after contrast (p = 0.009), tumour located in the left lobe (p = 0.02) and biliary ductal dilation (p = 0.027). An H-MCNs risk predictive nomogram was constructed, which showed excellent discrimination (areas under the receiver operating characteristic curve were 0.940) and consistent calibration between the predicted probability and actual probability.

**Conclusion:**

Among patients with H-MCNs, the location of the tumour, enhancement in CT scan, and biliary duct dilation are significantly independent risk factors. The appropriate treatment of H-MCNs is radical resection. Using our Nomogram could facilitate screening and identification of patients with liver cystic lesions.

## Background

With the development of the radiography technique and the increasing frequency of routine health examination, more and more liver cystic lesions have been discovered throughout these years. It was reported that about 20% of ordinary people have liver cystic lesions [1]. The most majority of cystic lesions are hepatic simple cysts (HSC), a kind of common benign cysts of the liver. HSC grows up by inches, and most of the HSC require no treatment or just fenestration [2]. However, there are other two more aggressive lesions in the liver, called intrahepatic biliary cystadenomas (IBC) and intrahepatic biliary cystadenocarcinoma (IBAC). IBC and IBAC are estimated to occupy less than 5% of the liver cysts [3, 4]. IBC was first reported by Henter et al. in 1887 [5]. Then in 1958, Emre described IBC as a liver cystic neoplasm with the pathological feature of “ovarian-like stroma(OS).”[6] By the year of 2010, the World Health Organization (WHO) redefined that the OS is the requirement for diagnosing cystadenoma both in liver and pancreas, and rename the IBC and IBAC as hepatobiliary mucinous cystic neoplasms (H-MCNs) [7, 8].

Although the equipment of imaging diagnosis has been improving quite a lot, such as contrasted-enhanced ultrasound (US), computed tomography (CT) and magnetic resonance imaging (MRI), the differential diagnosis for H-MCNs and HSC was still a big challenge [9]. IBC has a malignant potential to transform into IBAC in 20–30% patients, and the recurrent rate is much higher than HSC after fenestration therapy [10]. Because the biological characteristics and treatment strategies between H-MCNs and HSC are quite different, the accurate preoperative diagnosis of H-MCNs is of great significance. This study aimed to present our experience in the diagnosis and management of H-MCNs under the new WHO classification. Meanwhile, we developed and validated a predictive model to enhance the diagnostic accuracy between H-MCNs and HSC, and display the model with a nomogram.

### Methods

**Fig. 1:**
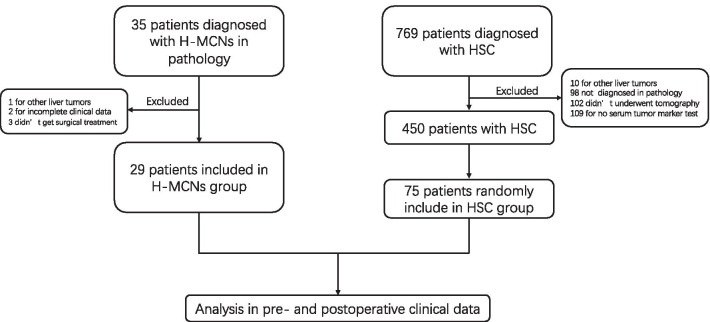
Flowchart of patient selection

From June 2011 to June 2019, a total of 29 patients underwent surgical treatment and pathologically diagnosed as H-MCNs within the WHO 2010 new classification standard at Zhejiang University, School of medicine, Sir Run-Run Shaw Hospital. All of the patients in this study underwent a routine pathological examination of surgical specimens. The pathological diagnosis was concluded by at least two experienced pathologists. Besides, 75 patients pathologically diagnosed as HSC between June 2011 and June 2019 were randomly included to compare the characteristics with the H-MCNs. The patients with other liver neoplasms, missing or incomplete data, nonoperative treatment, or these who weren't diagnosed in histology were excluded from the study (Fig. 1). Patients with liver cystic lesions in this cohort were routinely recommended to undergo the US or CT examination. For patients with unclear diagnosis in US or CT were recommended to underwent MRI examination.

Clinical and demographic variables, including age, gender, clinical symptoms, duration of symptoms, serum tumour markers, liver function, were obtained from medical records. Information about the preoperative imaging findings of US, CT, and MRI was reviewed. Radiological features like enhancement after contrast, multilocular cyst, septa, calcification, biliary ductal dilatation were collected. The surgical approaches, operation time, and bleed loss were reviewed from operation records. Tumour size was obtained from the pathological reports, which is defined as the longest diameter of the tumour. If the size was not mentioned in pathological reports, we referred to the most recently preoperative cross-sectional imaging report. The presence of OS was the requirement to diagnosis the H-MCNs in histology. This retrospective study was approved by the Ethics committee of Sir Run Run Shaw Hospital (Reference number SRR20200210).

Continuous variables were displayed as the mean ± standard deviation or medians with ranges. Categorical variables were presented as numbers and percentages. We used student’s t-test or Mann–Whitney U test to compare continuous variables, and chi-square or Fisher’s exact test to compare categorical variables among different groups. The subgroup was set by the cut-off value according to the receiver operating characteristic curve analysis reported by Wang et al.[11]

The univariate analysis was used to analyze the relationship of preoperative clinical characteristics and imaging features between HSC and H-MCN. Variables with statistical significance were further enrolled in the multivariate logistic analysis. We incorporated the variables significantly independent in multivariate logistic analysis to construct a preoperative H-MCNs risk prediction nomogram. And we forced factors that have great clinical relevance back into the model. Bootstraps with 1000 resamples were used to validate the calibration of the Nomogram. And we used receiver operating characteristic curve analysis and concordance Index (C-index) to assess the model discrimination. A P-value of less than 0.05 was deemed as statistical significance. All of the statistical analysis was conducted by SPSS (ver 23.0, USA) and R software (ver 3.5.3, USA).

### Result

**Fig. 2:**
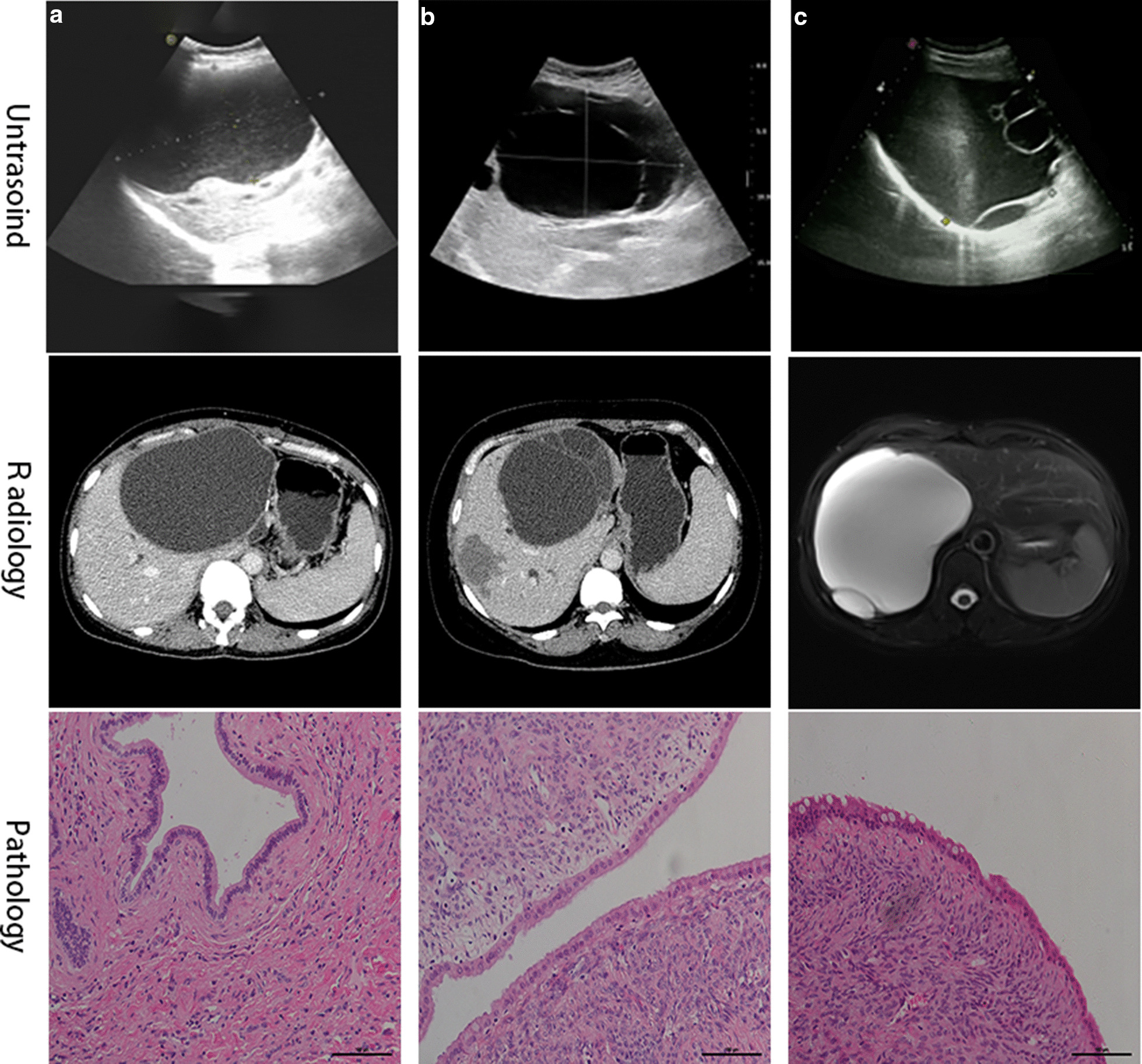
Photos of contrast CT, MRI, and the pathological section of patients with hepatobiliary mucinous cystic neoplasm (H-MCNs). (Heamatoxy-lin & eosin, × 40) Patient A was a 40–50 years old patient who discovered the liver cystic lesion with no symptoms for 8 years. Three years ago, she underwent laparoscopic left lateral hepatic lobectomy in her local hospital. But two years later, she was found a 17 cm cystic tumor in the liver again. We performed the laparoscopic left hemihepatectomy and caudate lobectomy for her. Patient B was a 31–40 years old patient who discovered “hepatic simple cyst” for 10 years. Two years ago, she was diagnosed with hepatic simple cyst and underwent liver cyst fenestration in another hospital. The postoperative pathological report showed the lesion was H-MCNs but wasn’t performed further treatment. She was admitted to our hospital for abdominal pain for two months. The US, CT images were showed above. We performed a laparoscopic liver cystic tumor resection and T-tube drainage for her. Patient C was a 51–60 years old patient admitted to our hospital due to being discovered a liver cystic lesion for ten years. Two years ago, she was performed fenestration, but the cyst still grew up gradually. By MRI picture, we could see a gaint cyst in the right lobe with septa. The corresponding histology (Haematoxylin and eosin staining) pictures showed OS and mucinous epithelial lining. All of those three patients were alive with no recurrence when the latest followed up

From June 2011 to December 2018, 29 patients diagnosed as H-MCNs and underwent surgical treatment in our institution, including 4 cases of IBAC. The images of US, CT, MRI, and corresponding histology were displayed in Fig. 2. Their demographics and clinical characteristics were presented in Additional file 1. The median age of the patients was 53 years (range, 47–62). A majority of patients were female (69%). About half of the patients initially complained about abdominal pain (48.3%); the median duration of symptoms was 48 months (range, 0.3–240 months). Interestingly, although the left part of the liver is much smaller than the right, most of the lesions (79.3%) were located on the left lobe.

Preoperative image examination and treatment characteristics between H-MCNs and HSC were shown in Table 1. Although most of the patients with H-MCNs underwent US, only 1 (3.4%) patient was accurately diagnosed by the US. Whereas, among the patients with HSC, the accuracy rate of the US was 90.4%. In the H-MCNs group, 26 and 14 patients respectively underwent CT and MRI, but only 12 (46.1%), and 8 (57.1%) of the patients were accurately diagnosed as H-MCNs by CT and MRI. The diagnostic accuracy of CT and MR was much higher in the patients with HSC. In our institution, half (50%) of the H-MCNs were performed a laparoscopic operation, and all (100%) of the patients with HSC underwent laparoscopic surgery. About the type of surgery, the operative intervention included cyst resection (34.5%) and partial liver resection (65.5%); 3 patients were initially performed fenestration, then concerted to radical operation because intraoperative frozen section examination suggested that the lesion may be H-MCNs. Compared with the H-MCNs, most of the patients with HSC (96%) underwent fenestration; 3 patients (4%) were performed radical resection due to misdiagnosing as H-MCNs. A total of 4 patients (14.2%) with H-MCNs and one patient with HCS suffered a postoperative complication.

**Table 1:** Image examination and treatment between hepatobiliary mucinous cystic neoplasms and hepatic simple cyst

	H-MCNs (n = 29)	HSC (n = 75)	*p* value
Preoperative imaging, x/n^†^ (%)			
Ultrasound (US)	1/29 (3.4)	66/73 (90.4)	< 0.001
Contrast computed tomography (CT)	12/26 (46.1)	62/67 (92.5)	< 0.001
Magnetic resonance imaging (MRI)	8/14 (57.1)	6/8 (75)	< 0.001
Operation, n (%)			< 0.001
Fenestration	0 (0)	72 (96)	
Tumor resection	10 (34.5)	2 (2.7)	
Partial liver resection	19 (65.5)	1 (1.3)	
Surgical approach, n (%)			< 0.001
Laparoscopy	14 (48.3)	75 (100)	
Open	15 (51.7)	0 (0)	
Estimated blood lost, median (IQR)	200 (100–300)	10 (5–13.75)	< 0.001
Operation time, median (IQR)	169 (213–288)	55 (45–90)	< 0.001
Complication, n (%)	5 (20)	3 (4)	0.037
Abdominal bleeding	1 (3.6)	0 (0)	
Bile leakage	2 (7.1)	0 (0)	
Incisional infection	1 (3.6)	0 (0)	
Pleural effusion	0 (0)	1 (1.3)	
Abdominal collection	0 (0)	1 (2.6)	
Incision infection	1 (3.6)	0 (0)	

*H-MCNs* hepatobiliary mucinous cystic neoplasms, *HSC* hepatic simple cyst

^†^X: diagnose accurately by the equipment; n: the number of the patient who have done the examination

During the study period, a total of 104 patients who had liver cyst lesions and underwent surgical treatment were included. Preoperative demographics and clinical characteristics of H-MCNs and HSC were displayed in Table 2. Twenty-three variables were used to be tested as potential predictors of H-MCNs in liver cystic lesions. In univariate analysis, eight preoperative variables including abdominal pain (*p* = 0.024), fever (*p* = 0.005), duration of the symptom (*p* < 0.001), serum CA19-9 elevation (*p* = 0.001), enhancement after contrast (*p* < 0.001), biliary ductal dilation (*p* < 0.001), septa (*p* = 0.002), location of the cyst (p < 0.001) were significantly different between the group of H-MCNs and HSC. These statistically significant factors were included in further analysis by a multivariable logistic regression model. On multivariable analysis (Table 3), with the reported as odds ratio (95%CI), enhancement after contrast (12.1[2.11–100]), lesion located in the left of the liver (11.0[1.71–121]), biliary ductal dilatation (9.80[1.46–92.2]) were independently associated H-MCNs. Although serum CA19-9 level was increased in many patients with H-MCNs, but the difference was not statistically significant (5.21[1.02–31.9], p = 0.053) in multivariable logistic regression analysis. In consideration of improving the sensitivity of the model, we also included the CA19-9 as a significant factor.

**Table 2:** Clinical and imaging feature of the patients with the hepatic liver lesion

	H-MCNs (n = 29)	HSC (n = 75)	*p*-value
*Demographics*			
Age, mean(SD), year			0.068
> 60	9 (31.0)	40 (53.3)	
≤ 60	20 (79.0)	35 (46.7)	
Sex, n (%)			0.463
Female	20 (69.0)	57 (76.0)	
Male	9 (31)	25 (24)	
Symptom, n (%)			
Abdominal pain	14 (48.3)	19 (25.3)	0.024
Abdominal fullness	7 (24.1)	10 (13.3)	0.181
Fever	4 (13.8)	0 (0)	0.005
Jaundice	2 (6.9)	0 (0)	0.076
Weight loss	1 (3.4)	0 (0)	0.275
Duration of the symptom (month)	1 (0.133–240)	60 (0.1–360)	< 0.001
Liver function			
AST (IQR), U/l	23 (13–38)	20 (18–23)	0.273
ALT (IQR), U/l	20 (13.5–31.5)	17 (14–22)	0.101
Total bilirubin (IQR), mg/dl	13.4 (11.2–16.2)	12.3 (10–16)	0.730
Serum tumor markers			
CA 19–9 (U/mL), n (%)			0.001
> 20	18 (64.3)	15 (27.3)	
≤ 20	10 (35.7)	40 (72.7)	
CEA (ng/mL), n (%)			0.469
> 5	2 (7.1)	2 (2.7)	
≤ 5	26 (92.9)	54 (72.0)	
CA 12–5 (U/mL), n (%)			0.117
> 20	6 (20.7)	5 (9.1)	
≤ 20	22 (75.9)	50 (90.9)	
AFP (ng/ml), n (%)			0.598
> 10	2 (7.1)	2 (3.6)	
≤ 10	26 (92.9)	54 (96.4)	
Imaging feature^†^, n (%)			
Biliary ductal dilation	17 (58.6)	4 (5.3)	< 0.001
Multilocular cyst	17 (58.6)	26 (83.9)	0.109
Enhancement after contrast	20 (69.0)	11 (14.7)	< 0.001
Septa	13 (44.8)	12 (16.0)	0.002
Calcification	2 (6.9)	12 (16.0)	0.223
Cyst size (cm), median (IQR)	8.5 (3.7–12.3)	9.1 (7.8–11.0)	0.131
Location of the cyst, n (%)			< 0.001
Left	23 (79.3)	22 (29.2)	
Right	3 (10.3)	39 (58)	
Both	3 (10.3)	14 (18.7)	

^†^Imaging features were collected based on abdominal contrast CT or abdominal contrast MRI

**Table 3:** Multivariate statistical analyses of Clinical characteristics in HSC and H-MCNs

	OR (95%CI)	p-value
Abdominal pain	3.71 (0.572–29.8)	0.178
Fever	NA	0.995
Duration of the symptom	0.97 (0.985–1.00)	0.600
CA199	5.21 (1.02–31.9)	0.053
Enhancement after contrast	12.1 (2.11–100)	0.009**
Intrahepatic location		
Right	–	–
Left	11.0 (1.71–121)	0.020*
Both	7.56 (0.871–108)	0.087
Biliary ductal dilation	9.80 (1.46–92.2)	0.027**
Septa	2.89 (0.455–20.3)	0.260

*OR* odds radio, *CI* confidence interval

Then, we conducted collinearity diagnosis for these independent risk factors, the variance inflation factor was 1.11, 1.03, 1.20, and 1.06, respectively, which means that there is no collinearity among these four factors, and the model composed of these four variables would be robust.

**Fig. 3:**
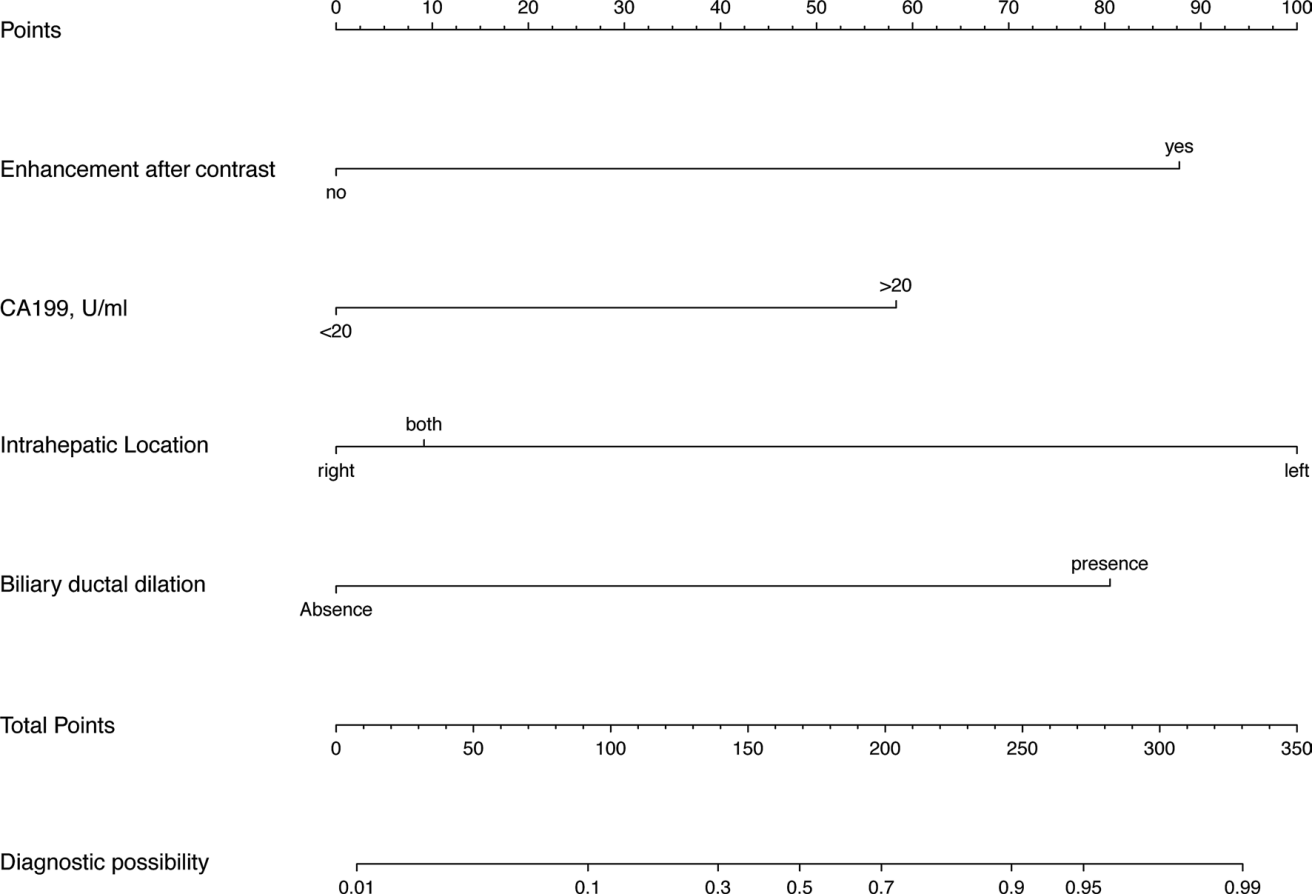
A nomogram to estimate the possibility of H-MCN in liver cystic lesions. To use this Nomogram, find the position of the variables on the corresponding axis, draw a vertical line to the points axis to get the points of the variable. Calculate the total points of all variables and draw a line from the corresponding total points axis to the possibility axis to get the probability of the H-MCNs

Based on the result of multivariate logistic regression analysis, these four individual predictive factors were incorporated in a preoperative prediction model. We display the model as a preoperative H-MCNs risk prediction nomogram (Fig. 3).

**Fig. 4:**
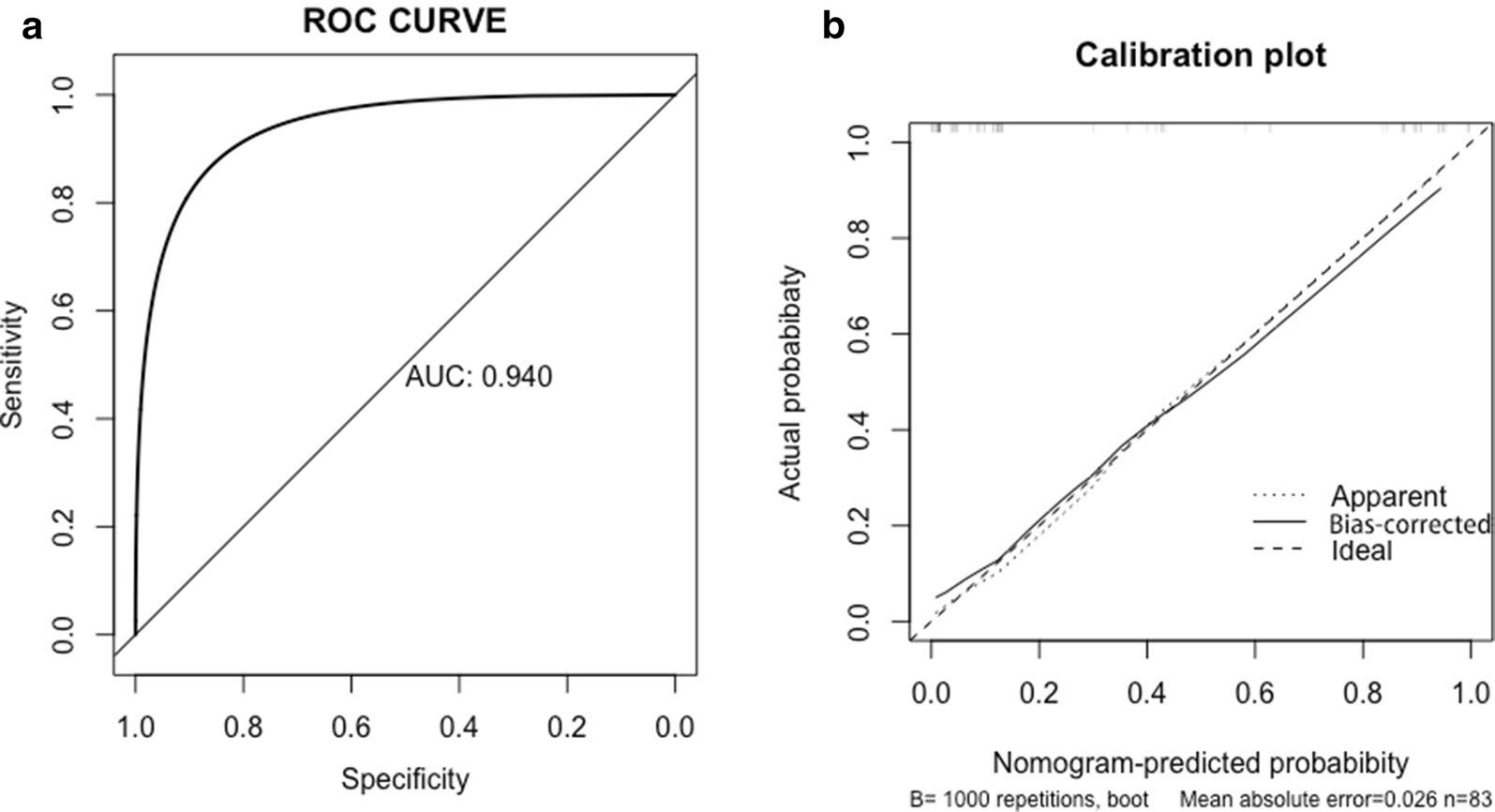
**a** ROC curve of the H-MCNs prediction nomogram. The ROC curve of the model showed excellent discrimination (C-index = 0.940). **b** Calibration plot for predicting the probability of H-MCNs in the hepatic cystic lesion. The predicted probability of the Nomogram was good agreement with the actual probability

To validate the discrimination of the prediction model, we created a ROC curve of the Nomogram (Fig. 4a), and displayed a C-index of 0.940, suggesting that the prediction has excellent discrimination. The calibration validation of the model was conducted by the calibration plot by bootstrapping with 1000 resamples (Fig. 4b). The predicted probability of the Nomogram was good agreement with the actual probability.

### Discussion

IBC and IBAC are kinds of rare liver cystic tumours, occupying only about 5% of liver cystic lesions [4]. In 2010, IBC and IBAC were redefined as H-MCNs by WHO new classification according to the presence of an ovarian-like stoma (OS) [7, 8]. About 20% of BAC tends to transform into IBAC. Thus, the treatment of H-MCNs should be more radical, and the accurate diagnosis of H-MCNs and HSC is of great significance. However, due to the lack of specific biochemical markers and imaging features, the identification of H-MCNs was still challenging. To improving the differential diagnosis between H-MCNs and HSC, previous researchers make their effects on finding some specific markers. [9, 12–14]. Nevertheless, these small-sample studies can hardly find significant differences between HSC and H-MCNs. Labib et at. reported that H-MCNs was often found as a single cyst (*p* = 0.006) [13]; Koffron et at. reported that the elevation of CA19-9 and CEA level in the cystic fluid were significantly different;[14] while Choi et al. found that cystic fluid analysis was useless to diagnose H-MCNs [9]. At present, the imaging examination is still the most effective method to diagnose H-MCNs, although lacking reliably specific image feature [10]. Frozen section after the fenestration is also an accurate method of diagnosing H-MCN, but it would be a risk to perform such a technique if the lesion harboured a carcinoma focus. In a word, the data on the preoperative diagnosis of H-MCNs remains scarce, and few studies were based on the new classification of H-MCNs [4–6, 8, 10, 15, 16].

In our cohort, a total of 29 patients diagnosed as H-MCNs with the presence of OS in pathology were reviewed. Major of H-MCNs occurred in women (20/29, 69%), particularly younger age women (≤ 60 year,79%), which is consistent with the previous study, although it didn’t reach significant differences [9, 13]. There are many studies compared the tumour markers between HSC and H-MCNs [9, 13, 14, 17]. In our study, we found that serum CA19-9 level was increased in many patients with H-MCNs, but the difference was not statistically significant (*p* = 0.053). Cyst fluid tumour markers analysis of H-MCNs remains controversial and needs more extensive sample sizes study to verify [9, 13]. Radiographic examination was still the primary tool to diagnose H-MCNs [18]. However, we revealed that only about half of patients with H-MCNs could be accurately diagnosed by the CT (12/26,46.1%) and MRI (8/14,57.1%), while the US was nearly no help to diagnose H-MCNs (1/29,3.4%). (Table 1) The imaging characteristics of H-MCNs, reported previously, were solitary, large, thick-wall, multilocular cystic with internal septation and calcification [11, 18]. Nevertheless, the imaging finding of many H-MCNs were are atypical, and some HSCs may also present these features, which cause great difficulty of diagnosis.

In multivariate analysis, three factors, including biliary ductal dilation, enhancement in CT and intrahepatic location, were significantly associated with H-MCNs. H-MCNs originate from congenitally aberrant bile ducts, and the cyst fluid is secreted by these abnormal bile duct cells. Compared with HSC, there is more blood supply in the wall, nodules and septa of H-MCNs. AS a result, the H-MCNs was often combined with bile duct dilatation and enhanced after contrast in imaging. What’s interesting was that although the normal right lobe of the liver is much bigger than the left, most lesions of H-MCNs (23/29,79.3%) located in the left lobe and was statistically significant in multivariate analysis. Zen et al. and Albores-Saavedra et al. found a similar phenomenon [19, 20]. In our view, this may because the high degree of left liver freeness, the tumour is prong to outward compression growth in the loose tissue, the mechanism need to be further explored.

The preoperative distinguish of H-MCNs and HSC was crucial to make the surgical plan. Because of the high rate of recurrence and premalignant progression, H-MCNs were recommended to undergo radical resection, such as complete cyst resection or anatomical liver resection, even liver transplantation [10, 21, 22]. It was reported that complete cyst resection could bring to long-term survival and a low rate of recurrence [10, 23]. According to a multi-institutional study, if the H-MCNs were performed fenestration, the recurrence could reach 48.6%, and might enhance the risk of malignant-transformation [10]. The treatment of large HSC is usually laparoscopic fenestration or unroofing when patients have the symptom of compression [25]. As a result, several HSC misdiagnosed as H-MCNs may undergo radical excision to suffer unnecessary surgical trauma.

Since the preoperative diagnosis of H-MCNs remains quite tricky, it is essential to establish a preoperative diagnosis model to enhance the differential ability of H-MCNs and HSC. We created a diagnosis nomogram that facilitates accurate identification and screening of H-MCNs preoperatively. Nomogram is a visualized tool that could vividly display the logistic regression model and help to make clinical decisions. In our Nomogram, three factors (Enhancement after contrast, intrahepatic location, and biliary dilation) were significantly associated with H-MCNs in the multivariate logistic regression analysis were included. Besides, serum CA19-9 level was widely reported to associate with H-MCNs in previous studies [10, 24, 25]. In our analysis, serum CA19-9 level was increased in many patients with H-MCNs, but the difference was not statistically significant in multivariate analysis (p = 0.053). In consideration of improving the sensitivity of the diagnostic model, we also included the CA19-9 as a significant factor. This is the first Nomogram of H-MCNs in liver cystic lesions. The advantages of the Nomogram were that it provides individualized risk assessment in a dynamic manner, and all the factors included are routinely reached in clinical practice. By utilizing this Nomogram, the patients with a high probability of H-MCNs would be recommended to undergo a complete resection. And some preoperatively invasive tests like biopsy or fluid aspiration would be replaced by using the Nomogram to get a risk probability.

There were some limitations to this study. First of all, the current study was a retrospective study with some inevitable selection bias. Second, although the H-MCNs were a rare disease, the sample size of the present study was too small and we have no ability to include enough samples for impeccable logistic regression. Thus, the Nomogram based on these 29 positive cases may not fully reflect the reality, and large-sample multicenter studies were still expected in the future. Before the wide acceptance of this Nomogram, it is desirable to validate this result from other centres. Third, considering whether the cystic fluid tumour marker level could help in the diagnosis of H-MCNs remains controversial, and not all of the liver cystic patients were routinely performed aspiration paracentesis, we didn’t include fluid tumour marker level as a predictive factor. Meanwhile, other specific markers understudy might further improve the accuracy of the prediction model of H-MCNs.

## Conclusion

H-MCNs were rare cystic tumour of the liver, which are difficulty in diagnosis. Enhancement after contrast, located in the left lobe of the liver and biliary ductal dilation, are significantly independent predictors to distinguish between H-MCNs and HSC. The H-MCNs risk predictive nomogram provides an accurate probability of H-MCNs risk in liver cystic lesions and helps to make the clinical decision.

## Supplementary Information

**Additional file 1: Table.** Characteristic and clinical feature of the patients with hepatobiliary mucinous cystic neoplasm (H-MCNs).

## Data Availability

We declared that materials described in the manuscript, including all relevant raw data, will be freely available to any scientist wishing to use them for non-commercial purposes, without breaching participant confidentiality. The data is available from the corresponding author.

## Abbreviations

H-MCNs: Hepatobiliary mucinous cystic neoplasms
HSC: Hepatic simple cyst
IBC: Intrahepatic biliary cystadenomas
IBAC: Intrahepatic biliary cystadenocarcinoma
OS: Ovarian-like stroma
WHO: World Health Organization
US: Ultrasound
CT: Computed tomography
MRI: Magnetic resonance imaging
C-index: Concordance index
