# Supplementary material for: Differentiation and management of hepatobiliary mucinous cystic neoplasms: a single centre experience for 8 years
Source: BMC Surg. 2021 Mar 20;21:146. doi: 10.1186/s12893-021-01110-9 (PMC7981987; doi:10.1186/s12893-021-01110-9)
Supplement: Supplementary file 1 — Additional file 1: Table. Characteristic and clinical feature of the patients with hepatobiliary mucinous cystic neoplasm (H-MCNs). [file 12893_2021_1110_MOESM1_ESM.docx]

**Additional table**. Characteristic and clinical feature of the patients with hepatobiliary mucinous cystic neoplasm (H-MCNs).

| Id no. | Age | Sex | Symptom | Duration(month) | Location | Operation ^†^ | Pathology ^‡^ | Tumor size（cm） |
| --- | --- | --- | --- | --- | --- | --- | --- | --- |
| 1 | 61-70 | 1 | No symptom | 48 | left | CR | H-MCNs | 13 |
| 2 | 51-60 | 1 | Abdominal pain | 120 | left | LR | BCAC | 16 |
| 3 | 61-70 | 2 | Abdominal pain | 1 | left | LR | H-MCNs | 8.1 |
| 4 | 41-50 | 1 | No symptom | 0.5 | left | CR | H-MCNs | 7.8 |
| 5 | 21-30 | 1 | Abdominal fullness | 1 | left | CR | H-MCNs | 13.1 |
| 6 | 61-70 | 2 | No symptom | 6 | left | CR | H-MCNs | 10.8 |
| 7 | 41-50 | 1 | Abdominal pain | 6 | left | LR | H-MCNs | 4.85 |
| 8 | 61-70 | 1 | Abdominal pain | 240 | left | LR | BCAC | 8.4 |
| 9 | 51-60 | 1 | Abdominal pain | 0.5 | left | LR | BCAC | 8.98 |
| 10 | 51-60 | 1 | Abdominal pain | 3 | left | LR | H-MCNs | 6.78 |
| 11 | 41-50 | 2 | Abdominal pain | 0.133 | right | LR | H-MCNs | 8.9 |
| 12 | 41-50 | 1 | Abdominal pain | 0.333 | right | LR | H-MCNs | 12.1 |
| 13 | 51-60 | 2 | No symptom | 0.7 | left | LR | H-MCNs | 15.2 |
| 14 | 51-60 | 1 | No symptom | 1 | Left and right | CR | H-MCNs | 7.7 |
| 15 | 21-30 | 1 | No symptom | 12 | left | CR | H-MCNs | 12 |
| 16 | 71-80 | 1 | Abdominal pain | 48 | left | LR | H-MCNs | 9.1 |
| 17 | 41-50 | 1 | No symptom | 0.5 | left | LR | H-MCNs | 9.5 |
| 18 | 71-80 | 1 | Abdominal pain | 0.5 | left | LR | H-MCNs | 9.9 |
| 19 | 51-60 | 1 | Abdominal pain | 4 | left | LR | H-MCNs | 19 |
| 20 | 51-60 | 1 | Abdominal pain | 0.333 | left | LR | H-MCNs | 8.3 |
| 21 | 51-60 | 2 | Early satiety | 0.3 | left | LR | H-MCNs | 10.3 |
| 22 | 81-90 | 2 | No symptom | 0.5 | Left and right | CR | BCAC | 8.1 |
| 23 | 51-60 | 2 | Abdominal pain | 2 | left | CR | H-MCNs | 16 |
| 24 | 41-50 | 1 | No symptom | 0.333 | left | LR | H-MCNs | 24 |
| 25 | 61-70 | 2 | Early satiety | 0.5 | left | CR | H-MCNs | 7.4 |
| 26 | 61-70 | 2 | Fever | 240 | Left and right | LR | BCAC | 8.9 |
| 27 | 41-50 | 1 | No symptom | 120 | left | LR | H-MCNs | 11.5 |
| 28 | 31-40 | 1 | Abdominal pain | 48 | left | CR | H-MCNs | 10 |
| 29 | 51-60 | 1 | Abdominal fullness | 84 | right | LR | H-MCNs | 13 |

†: H-MCNs：hepatobiliary mucinous cystic neoplasms; BACA: intrahepatic biliary cystadenocarcinoma.

‡: CR: complete cyst resection; LR: partial liver resection
